# Factors Influencing Hypoglycemia in Type 2 Diabetes Mellitus Outpatients with State Health Insurance at Regional General Hospitals in Jakarta, Indonesia

**DOI:** 10.2174/0115733998280552231228064154

**Published:** 2024-01-11

**Authors:** Putu Rika Veryanti, Rani Sauriasari, Ratu Ayu Dewi Sartika, Berna Elya

**Affiliations:** 1 Faculty of Pharmacy, Universitas Indonesia, Depok, Indonesia;; 2 Faculty of Public Health, Universitas Indonesia, Depok, Indonesia

**Keywords:** Influencing factors, hypoglycemia, type 2 DM, outpatient, JKN health insurance

## Abstract

**Background:**

Hypoglycemia is an acute episode that can lead to death in patients with diabetes mellitus (DM). This condition is preventable with patient education, and identifying factors influencing their occurrence is essential to creating effective and efficient education. It also leads to prevention and control by re-organizing the service system and diabetes policies. This study aimed to determine factors contributing to hypoglycemic episodes in type 2 DM outpatients covered by the state-provided *Jaminan Kesehatan Nasional* (JKN) health insurance.

**Methods:**

The study used a cross-sectional design and collected data from five regional general hospitals in Jakarta, Indonesia. The outpatients were sampled consecutively from two hospitals in September–November 2021, one in January–March 2022, and two others in April–June 2023. Interviews produced primary data related to experienced hypoglycemic episodes, and medical records provided secondary data on patients' clinical characteristics and treatments. Binary logistic regression analysis was employed to process the contributing factors statistically.

**Results:**

From 501 patients who met the inclusion and exclusion criteria, it was found that the prevalence of hypoglycemia was 53.3%. Factors that significantly increased hypoglycemic risk (*p* < 0.05) were high HbA1c levels (OR 1.9; 95% CI 1.2–2.9), comorbidities (OR 1.6; 95% CI 1.1–2.4), insulin/sulfonylurea therapy (OR 2; 95% CI 1–4), non-smoking habit (OR 2.2; 95% CI 1.3–3.6) and physically active lifestyle (OR 1.8; 95% CI 1.2–2.6).

**Conclusion:**

The prevalence of hypoglycemia in type 2 diabetes mellitus (DM) outpatients with the state-provided health insurance *Jaminan Kesehatan Nasional* (JKN) at general hospitals in Jakarta is high. The diabetes self-management education (DSME) services provided by health professionals for these outpatients must be further improved.

## 1 INTRODUCTION

The prevalence of diabetes mellitus (DM) worldwide was estimated to reach 537 million adults (aged 20-79 years) in 2021 and is projected to increase to 643 million in 2030 and 783 million in 2045. The prevalence of DM is consistently increasing in several countries, including Indonesia. Indonesia had the fifth-highest number of people with DM worldwide in 2021 [1]. The Indonesia Basic Health Research 2018 reported that 20.4 million, or 8.5% of the population, were diagnosed with DM, dominated by those younger than 65 [2]. This figure was estimated to multiply in the future. The World Health Organization (WHO) predicted that in 2030, Indonesia will have 2.5 times more people suffering from DM than in the previous three decades [1].

The government has introduced numerous measures to control DM, including the state-provided health insurance *Jaminan Kesehatan Nasional* (JKN) to promote better healthcare services [3, 4]. On the one hand, with the health insurance financing system and based on the principle of cooperation, the people can benefit from this program. However, on the other hand, the program policy imposes restrictions on drug use in patients, which might cause the algorithm for type 2 DM therapies to deviate from the international consensus recommendation [5]. For instance, there are several differences between the management of type 2 DM in Indonesia [6] and the latest recommendations from the American Diabetes Association (ADA) and the European Association for the Study of Diabetes (EASD) in 2018 [7].

ADA/EASD recommends metformin with a combination of GLP-1 antagonists/SGLT2 inhibitors as first-line therapy for type 2 DM with the presence of comorbidities: atherosclerotic cardiovascular disease (ASCVD), heart failure, and impaired kidney function [7]. In contrast, Indonesia has not added GLP-1 antagonists and SGLT2 inhibitors to the national formulary. It means consent from patients or their families should be obtained before prescribing the drugs, for they will be responsible for the total costs. As an alternative, insulin with proven cardiovascular safety is considered for combined treatment with metformin [6]. Another difference is in the intensification of insulin injection. The Indonesia algorithm recommends adding insulin for type 2 DM patients who do not achieve the therapy target with three-oral drug combinations. If this fails to attain the expected target, only a GLP-1 antagonist will be added to the treatment. Insulin can be injected when HbA1c > 9% and symptoms of catabolism are present. ADA/EASD recommends the opposite: If the triple-combination therapy is ineffective, injecting GLP-1 agonists remains the preferred option to insulin. Insulin can be injected when HbA1c > 11% or symptoms of catabolism are present [7].

The above differences in patient preconditions for combination therapy result in more insulin users, especially in secondary and tertiary care in Indonesia [8, 9]. These policy differences make it essential to improve diabetes patient education by implementing Diabetes Self-Management Education (DSME) and Diabetes Self-Management Support (DSMS) [10, 11].

Besides insulin, sulfonylureas are also widely prescribed for type 2 DM patients in Indonesia. ADA/EASD recommends adding sulfonylurea (oral antidiabetic) to insulin as first-line combination therapy for low-income type 2 DM patients without a history of ASCVD and chronic kidney disease (CKD) [7]. Several studies reported that sulfonylurea and insulin strongly predict hypoglycemia in type 2 DM patients [12-14].

Hypoglycemia is an acute complication of diabetes that can lead to death [15, 16]. Many hypoglycemic episodes in type 2 DM patients are undetected. The cause might be patients not being well informed or aware of the signs [17-20]; thus, they are not reported and recorded in the healthcare system. It has been shown that hypoglycemia is associated with elevated morbidity and mortality rates [21-23]. This potentially leads to prolonged hospital stays, which inevitably affect the total cost incurred by patients and the government [24-26]. In 2016, the central government's total medical expenditure for managing hypoglycemia was considerably high, amounting to USD 23 million [27].

Even though it has severe impacts, hypoglycemia is preventable. This allows health workers to apply preventive measures, including improving patient education [28-30]. An individual approach catered to patient characteristics is needed for effective and efficient education [31]. Scientific information on any contributing factors of hypoglycemic episodes relevant to type 2 DM patients in Indonesia should be gathered to design this measure. However, hypoglycemia in type 2 DM outpatients with the state health insurance JKN remains underresearched.

Based on the description above, this study aimed to determine factors contributing to hypoglycemic episodes in type 2 DM outpatients covered by the state-provided health insurance *Jaminan Kesehatan Nasional* (JKN). In addition, health workers can use the study results as input to develop and deliver materials to effectively and efficiently educate diabetic patients. Knowing the risk factors of hypoglycemia can also lead to prevention and control by re-organizing the service system and diabetes policies.

## 2 MATERIALS AND METHOD

### 2.1 Study Design

This was a cross-sectional study. The data were collected using questionnaires and medical records.

### 2.2 Population and Sample

The research population was type 2 DM outpatients in five general hospitals in Jakarta. Participants were selected based on these inclusion criteria: they were diagnosed with type 2 DM, insured with JKN, had taken antidiabetic drugs for at least one year, and were willing to participate in the study. Meanwhile, those with gestational diabetes were excluded.

The sample size was estimated based on the following conditions: Assuming the prevalence of hypoglycemia in type 2 DM (P) is 50%; Z is a statistic corresponding to level confidence (1.96); with a 95% confidence level (95% CI); tolerated error (d) is 5%. The formula was as follows:

n = 
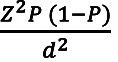
;

Notes:

n = Sample size

Z = Statistical corresponding to the level of confidence

P= Expected proportion of the population

d = Tolerated error

The study was conducted in all five general hospitals in Jakarta. The hospital service period was divided into 4 quarterly periods. Data collection in two hospitals was from September to November 2021, one hospital from January to March 2023, and two others from April to June 2023. One time period in each hospital was sampled using simple random sampling, and data were collected on all patients admitted during the sampling period.

### 2.3 Research Tools

In identifying hypoglycemia, we used a questionnaire that has previously been tested for validity and reliability. This questionnaire consists of 4 close-ended questions (yes/no) related to symptoms, actions taken, and documentation of blood glucose during hypoglycemia. This question refers to the classification of hypoglycemia according to the ADA (2013) [32]. Validity and reliability tests were carried out on 20 type 2 DM outpatients.

In the first question of the questionnaire, patients were asked to select the symptoms of hypoglycemia experienced in the last 3 months by ticking the column, such as excessive hunger, sweating, nausea, paleness, tingling, heart palpitations, restlessness, anxiety, trembling, weakness, dizziness, headache, confusion, seizures, loss of consciousness, and coma. The second question was whether the symptoms improved after consuming sweet foods or drinks, the third was whether the patient needed help from a health worker when experiencing hypoglycemia, and the last question was whether the patient had a blood glucose check when experiencing hypoglycemia. If yes, they were asked whether the sugar level was ≤ 70 dl or > 70 mg/dl. The answers to these questions were analyzed and categorized into the following 5 classifications of hypoglycemia (Table **1**) [32]:

If the patient answers “Yes” to questions number 1 and 3, then the patient is categorized as having severe hypoglycemia. If the patient answers “Yes” to questions number 1, 2, and 4 (blood glucose ≤ 70 mg/dl), then the patient is experiencing documented symptomatic hypoglycemia. If the patient only answers “Yes” to question 4 (blood glucose ≤ 70 mg/dl), then the patient is categorized as asymptomatic (documented) hypoglycemia. The probable symptomatic hypoglycemia category is experienced if the patient answers “Yes” to questions number 1 and 2. Finally, pseudo-hypoglycemia is experienced if the patient answers “Yes” to questions 1, 2, and 4. For patients who answer “No” to all questions, the patient does not experience hypoglycemia.

Data on factors influencing hypoglycemia were documented on a data collection form obtained from patient medical records. Data collected included personal characteristics, such as gender, age, BMI, and duration of DM; clinical characteristics, such as HbA1c, glomerular filtration rate (GFR), comorbidities, and treatment (insulin/SU user or not). Data related to lifestyle, such as smoking, alcohol, and physical activity, were obtained from patient interviews.

### 2.4 Data Collection

Researchers assisted by research assistants carried out data collection. Previously, the research assistant explained how the patient should complete the questionnaire and data collection form. In the data collection process, at first, type 2 DM patients were explained the research description and procedures for filling out the questionnaire. Patients who agreed to be involved in the research completed identification such as name, age, gender, address, and telephone number and signed the informed consent. Next, the patient filled out a questionnaire. When the questionnaires were collected, the research assistant confirmed that the questionnaire had been filled in completely and then interviewed the patient regarding lifestyle, such as smoking, consuming alcohol, and physical activity. Researchers and research assistants also completed the data collection form by filling in data on clinical characteristics and patient treatment from medical records. The variables recorded on the data collection form have been explained in sub-chapter 2.3. In completing the data collection form, we also confirmed with doctors and nurses regarding incomplete or unclear data in the medical record.

### 2.5 Data Analysis

All data in this study were explained with the help of IBM 25 statistical SPSS. Descriptions of personal characteristics, clinical characteristics, lifestyle, and treatment of patients were analyzed by univariate statistics and shown in tabular form. Factors influencing hypoglycemia were statistically analyzed using bivariate analysis (chi-square) to partially assess the relationship between patient characteristics and hypoglycemia. In bivariate analysis, we used OR and 95% CI. Variables with a *p*-value < 0.25 were input for the multivariate analysis. Binary logistic regression with backward elimination was used to determine the most influencing factors of hypoglycemia in the study participants. Adjusted odds ratio (AOR), *p*-value, and 95% confidence interval for each variable were also obtained from this analysis.

## 3 RESULTS

Screened with the predetermined inclusion and exclusion criteria, 501 type 2 DM outpatients with JKN health insurance participated in this study. During three months of observation, 53.3% of patients had experienced hypoglycemia. The distribution of hypoglycemia in type 2 DM outpatients with the state-provided health insurance JKN is shown in Table **2**.

The majority of patients (25.5%) were confirmed probable symptomatic hypoglycemia. They experienced typical symptoms of hypoglycemia, and the condition improved after consuming sweet foods/drinks, but this could not be verified by blood glucose determination. The blood glucose levels may fall to ≤ 70 mg/dl.

About 10.6% of patients experienced documented symptomatic hypoglycemia. Others (8.4%) experienced pseudo-hypoglycemia, 6,6% experienced asymptomatic documented hypoglycemia, and 2.2% of patients experienced severe hypoglycemia, which needed help from health workers to overcome it.

Table **3** shows the patient characteristics, antidiabetic, and lifestyle profile of type 2 DM outpatients with JKN health insurance. The majority of type 2 DM outpatients were female (57.7%), and 62.3% of patients were <60 years old. Regarding BMI, we used the overweight cut-off point for Asians (≥ 23 kg/m^2^), and 59.9% of patients had a BMI ≥ 23 kg/m2. 80.6% of patients were diagnosed with DM in less than 5 years.

The clinical characteristics profile showed that 71.1% of patients had controlled HbA1c, good kidney function (78.8%), and 70.7% had comorbidities. Based on antidiabetic use, 91.4% of patients were insulin/sulfonylurea users.

In the lifestyle category, we also found that 83.4% of type 2 DM patients do not smoke, 91.2% do not consume alcohol, and 55.9% are still physically active (55.9%).

The bivariate chi-square test found five independent variables with a *p*-value < 0.25: HbA1c level, comorbidities, antidiabetics, smoking, and physical activity. The bivariate analysis results are presented in Table **4**.

Based on Table **4**, it is known that patients with uncontrolled HbA1c (≥ 7%) are 2.11 times more at risk of hypoglycemia compared to patients with controlled HbA1c (<7%). The presence of comorbidities also puts patients 1.67 times more at risk of hypoglycemia than those who do not. From antidiabetic therapy, it was found that insulin/SU users were 2.28 times more at risk of hypoglycemia than non-insulin/SU users. Patients who do not smoke (OR 0.519; CI 95% 0.321-0.839) and have active physical activity (OR 1.848; CI 95% 1.294-2.642) are also known to be at greater risk of hypoglycemia than those who smoke and are less active.

After controlling for the effect of all variables, the multivariate analysis shows five factors that significantly influenced hypoglycemia among the participants (*p* < 0.05). Participants with high HbA1c levels (uncontrolled diabetes), the presence of comorbidities, insulin/sulfonylurea users, no smoking habits, and a physically active lifestyle are more at risk of experiencing hypoglycemia in this study, as shown in Table **5**.

Participants with high HbA1c (≥ 7%) were 1.9 times more likely to experience hypoglycemia than those with low HbA1C (< 7). At the same time, comorbidities caused a 1.6 times higher risk of hypoglycemia. As for the treatment, it was found that most participants received insulin or sulfonylurea therapy. Patients who received those therapy were 2 times at higher risk of hypoglycemia. In the lifestyle category, participants who smoked were less likely to experience hypoglycemia than those who did not smoke regularly (OR 0.45; CI 95% 0.28-0.77). While physically active participants had a 1.8 times higher risk of hypoglycemia than those with less physical activity.

## 4 DISCUSSION

The prevalence of hypoglycemia in type 2 DM outpatients with the state-provided health insurance *Jaminan Kesehatan Nasional* (JKN) is relatively high. Most of them experienced probable symptomatic hypoglycemia. They experienced typical symptoms of hypoglycemia.

Low blood glucose is characterized by autonomic and neuroglycopenic symptoms [16]. Autonomic symptoms can include sudden hunger, shaking, sweating, restlessness, increased heart rate, nausea, and vomiting. Autonomic symptoms can occur at sugar levels < 60 mg/dl, thereby activating the autonomic nervous system [33]. Meanwhile, neuroglycopenic symptoms are characterized by feelings of weakness, dizziness, headaches, blurred vision, and decreased consciousness. Patients with < 50 mg/dl of blood glucose can experience neuroglycopenic symptoms [34].

Biochemical documentation did not confirm those symptoms in probably symptomatic hypoglycemia because they do not have glucometers. They only check blood glucose during scheduled visits to healthcare facilities, whereas self-monitoring of blood glucose in DM patients, especially insulin users, is essential to prevent severe hypoglycemia [35, 36]. However, patients who have been well educated about diabetes take the initiative to check their blood glucose independently when experiencing hypoglycemia, either with a personal glucometer or immediately going to the nearest health service facility to check their blood glucose.

Hypoglycemia is associated with increased mortality and morbidity [37, 38]. It causes an increase in the length of hospital stay and ultimately impacts health costs incurred by patients and the government [39, 40]. State-provided health insurance *Jaminan Kesehatan Nasional* (JKN) data in 2016 shows that 18.7% of DM patients were hospitalized with complications of hypoglycemia. The total costs for hypoglycemia treatment reached US $ 23 million, comprising 71% for hospitalization, 26% for outpatient care, and the rest for non-diabetes-related medication [27].

Several preconditions related to the patient's clinical characteristics, antidiabetics, and lifestyle were identified as the significant influencing factors of hypoglycemia in this study. Hypoglycemia is influenced by HbA1c [41]. Au *et al.* explained that for every 1% increase in HbA1c, there was a 3% increase in non-severe hypoglycemic episodes in the last 30 days (95% CI 1%–6%). High HbA1c levels may indicate a large glycemic variability, which has been demonstrated as a factor of hypoglycemia in type 2 diabetic patients [42, 43].

Another factor that influences hypoglycemia in type 2 DM is comorbidities [44]. Au *et al.* discovered that comorbidities in type 2 DM patients could double the occurrence of non-severe hypoglycemia in the last 30 days (95% CI 53%–180%) [45]. Comorbidities that can induce hypoglycemia in type 2 DM include cerebrovascular disease [46], malignancy, heart failure [21], obesity [47], decreased cognitive function [48], and chronic kidney disease [49]. Decreased kidney function can reduce the clearance of antidiabetic drugs, resulting in drug accumulation in the blood and causing hypoglycemia [50]. Comorbidities in type 2 DM patients also increase the risk of polypharmacy [51-53], which potentially induces drug-drug interactions-a known cause of hypoglycemic episodes in these patients [54].

The use of insulin and sulfonylurea is also one of the predictors of hypoglycemia [55, 56]. Insulin was the treatment of choice for those with cardiovascular and renal comorbidities, including atherosclerotic cardiovascular disease (ASCVD), heart failure, and impaired kidney function [6]. This treatment differed from ADA/EASD, which recommends a combination of metformin and GLP-1 RA/SGLT2 inhibitors as first-line therapy for patients with these conditions [5]. Even though GLP-1 RA/SGLT2 inhibitors prove effective and safe for type 2 DM patients, in Indonesia, these drugs are relatively new and can thus be costly, mainly because they are not yet included in the national formulary; hence, uncovered by the JKN health insurance [5].

For the above reasons, insulin is still the preferred option for type 2 DM therapy in the country because it is cardiovascularly safe and relatively cheap compared to GLP-1 RA or SGLT2–inhibitors [57-59]. Supporting evidence has been discussed in a systematic review of 2351 studies, which revealed that basal insulin does not increase the risk of cardiovascular events or death in patients with type 2 DM [60]. In addition, a meta-analysis study also showed that insulin therapy did not affect cardiovascular events, heart failure, and mortality risk in these patients [61]. Apart from insulin, sulfonylurea therapy is also widely prescribed for type 2 DM patients with JKN health insurance. Sulfonylureas are first-line treatment, either as monotherapy or combined with metformin, in low-income patients without a history of ASCVD and chronic kidney disease [6, 7].

On the other hand, various studies have proven that SGLT2 inhibitors and GLI-1 RA in type 2 DM patients can reduce the risk of hypoglycemia [62, 63]. Diabetes Mellitus patients with comorbid chronic kidney disease (CKD) with a mean age of 73 years who used SGLT2 inhibitors / GLP-1 RA were less at risk of hypoglycemia when compared with sulfonylureas (adjusted HR, 0.30; 95% CI, 0.14-0.65) [64]. Other studies also show that using GLP-1 RA (except albiglutide) can significantly increase the risk of hypoglycemia compared to placebo. Still, the risk of hypoglycemia is lower when compared with insulin and sulfonylureas [62]. Besides its effectiveness in controlling patient blood glucose, SGLT2 inhibitor and GLP-1 RA have lower hypoglycemia side effects.

Policies regarding the use of insulin and sulfonylurea can increase the risk of hypoglycemia in type 2 DM patients. Based on the results of this study and various other studies worldwide, the Indonesian government should consider including SGLT2 inhibitors and GLP-1 RA in the national formulary.

Besides that, smoking [65] and physical activity [66, 67] are part of habits or lifestyles that can affect blood glucose management in diabetic patients [68-70]. Smoking substantially raises postprandial blood sugar levels, and nicotine in cigarettes can increase insulin resistance and hyperglycemia [71, 72]. Furthermore, with nicotine, the polycyclic aromatic hydrocarbon (PAH) content induces several enzymes in the liver: CYP (CYP1A1 and CYP1A2) and, possibly, CYP2E1 and UGT. As a result, the blood concentrations of antidiabetics and other drugs metabolized by these enzymes are decreasing due to increased drug metabolism and excretion, producing weaker effects than desired. Therefore, type 2 DM patients who smoke regularly require higher doses of antidiabetics to lower their blood glucose levels [73].

Most participants were in the productive age range, working and exercising regularly (at least 150 hours/week). High physical activity requires a lot of energy, which can lower blood glucose without adequate calorie intake [74, 75]. This result corresponds to a study in Canada, which found a higher risk of hypoglycemia among working type 2 diabetic patients than those with no occupation. Being employed was associated with a 45% increase (95% CI 1%-109%) in non-severe hypoglycemia in the past 30 days [45].

Other studies have also found other factors influencing hypoglycemia that may not have been found in this study. It could be due to patient characteristics, treatment therapy, or study design differences. The limitation of cross-sectional research is that it can only explore the primary relationship between variables without identifying causal relationships.

Another limitation of this study is that the identified hypoglycemic episodes were self-reported, which can lead to bias in the results. However, it was minimized by limiting the observation period, *i.e*., only hypoglycemic episodes in the last three months.

## CONCLUSION

The prevalence of hypoglycemia in type 2 diabetes mellitus (DM) outpatients with the state-provided health insurance *Jaminan Kesehatan Nasional* (JKN) at general hospitals in Jakarta was categorically high. Most of them experienced probable symptomatic hypoglycemia. Moreover, factors significantly influencing hypoglycemia were high HbA1c (uncontrolled blood glucose), no smoking habit, a physically active lifestyle, comorbidities, and insulin/sulfonylurea users.

## RECOMMENDATION

The results of this study can be used to evaluate and further improve the diabetes self-management education (DSME) health professionals provide for type 2 DM outpatients with JKN health insurance. Another recommendation is that the Indonesian government should consider including SGLT2 inhibitors and GLP-1 RA in the national formulary.

Future research is recommended to perform a prospective study on the prevalence of hypoglycemia in type 2 DM patients and analyze its influencing factors.

## Figures and Tables

**Table 1:** Classifications of hypoglycemia.

**No**	**Type of Hypoglycemia**	**Definition**
1	Severe Hypoglycemia	Patients need the help of health professionals to provide carbohydrates, glucagon, or other corrective measures.
2	Documented symptomatic hypoglycemia	The patient has typical symptoms of hypoglycemia and blood glucose ≤ 70 mg/dl.
3	Asymptomatic documented hypoglycemia	Patients with blood glucose ≤ 70 mg/dl but without typical symptoms of hypoglycemia
4	Probable symptomatic hypoglycemia	The patient experiences typical symptoms responsive to self-treatment but not confirmed by biochemical documentation but may be caused by a blood glucose ≤ 70 mg/dl.
5	Pseudo-hypoglycemia	Patients experience typical symptoms, blood glucose > or close to 70 mg/dl

**Table 2:** Distribution of Type of Hypoglycemia (n=501).

**Type of hypoglycemia**	**Number**	**Percentage**
**Hypoglycemia**	**267**	**53.3**
- Severe hypoglycemia	11	2.2
- Documented symptomatic hypoglycemia	53	10.6
- Asymptomatic documented hypoglycemia	33	6.6
- Probable symptomatic hypoglycemia	128	25.5
- Pseudo-hypoglycemia	42	8.4
**No hypoglycemia**	**234**	**46.7**

**Table 3:** Personal and clinical characteristics, antidiabetics, lifestyle profile of type 2 diabetes mellitus outpatients with the JKN health insurance (n=501).

**Variable**	**Number**	**Percentage**
**Sex**	-	-
Male	212	42.3
Female	289	57.7
**Age**	-	-
<60 years old	312	62.3
≥60 years old	189	37.7
**Body Mass Index**	-	-
<23 kg/m^2^	201	40.1
≥23 kg/m^2^	300	59.9
**Duration of DM**	-	-
<5 years	404	80.6
≥ 5 years	97	19.4
**HbA1c level**	-	-
<7%	356	71.1
≥7%	145	28.9
**Glomerulus Filtration Rate**	-	-
<60 ml/min/1.73 m^2^	106	21.2
≥60 ml/min/1.73 m^2^	395	78.8
**Comorbidity**	-	-
Yes	354	70.7
No	147	29.3
**Antidiabetics**	-	-
Insulin/sulfonylurea user	458	91.4
Not insulin/sulfonylurea user	43	8.6
**Smoking**	-	-
Yes	83	16.6
No	418	83.4
**Alcohol**	-	-
Yes	44	8.8
No	457	91.2
**Physical activity**	-	-
Physically active	280	55.9
Less physically active	221	44.1

**Table 4:** Bivariate analysis of factors influencing hypoglycemia in type 2 diabetes mellitus outpatients with the JKN health insurance (n=501).

**Variable**	**Hypoglycemic Episode**		**OR (95% CI)**	***p*-value**
	**Yes**	**No**		
**Sex**	-	-	-	-
Male	107 (50.47)	105 (49.53)	0.88 (0.576-1.172)	0.278
Female (Reference)	160 (55.36)	129 (44.64)		
**Age**	-	-	-	-
≥60 years old	106 (56.08)	83 (43.92)	1.2 (0.833-1.721)	0.330
<60 years old (reference)	161 (51.6)	151 (48.4)		
**Body Mass Index**	-	-	-	-
≥23 kg/m^2^	163 (54.3)	137 (45.7)	0.469 (0.247-0.890)	0.77
< 23 kg/m^2^ (reference)	104 (51.7)	97 (48.3)		
**Duration of DM**	-	-	-	-
≥5 years	54 (55.67)	43 (44.33)	1.126 (0.721-1.757)	0.601
<5 years (reference)	213 (52.72)	191 (47.28)		
**HbA1c Level**	-	-	-	-
≥7%	96 (66.21)	49 (33.79)	2.119 (1.419-3.165)	0.000*
<7% (reference)	171 (48.03)	185 (51.97)		
**GFR**	-	-	-	-
<60 ml/min/1.73 m^2^	56 (52.83)	50 (47.17)	0.977 (0.636-1.501)	0.914
≥60 ml/min/1.73 m^2^ (reference)	211 (53.42)	184 (46.58)		
**Comorbidity**	-	-	-	-
Yes	202 (57.06)	152 (42.94)	1.677 (1.138-2.470)	0.009*
No (reference)	65 (44.22)	82 (55.78)		
**Antidiabetics**	-	-	-	-
Insulin/sulfonylurea user	252 (55.0)	206 (45.0)	2.283 (1.188-4.390)	0.011*
Not insulin/sulfonylurea user (reference)	15 (34.9)	28 (65.1)		
**Smoking**	-	-	-	-
Yes	33 (39.76)	50 (60.24)	0.519 (0.321-0.839)	0.007*
No (reference)	234 (55.98)	184 (44.02)		
**Alcohol**	-	-	-	-
Yes	26 (59.09)	18 (40.91)	1.295 (0.691-2.427)	0.420
No (reference)	241 (52.74)	216 (47.26)		
**Physical activity**	-	-	-	-
Physically active	168 (60)	112 (40)	1.848 (1.294-2.642)	0.001*
Less Physically active (reference)	99 (44.8)	122 (55.2)		

**Note:** * = *p*<0.25, OR, Odds Ratio.

**Table 5:** Multivariate analysis of factors influencing hypoglycemia in type 2 diabetes mellitus outpatients with the JKN health insurance (n=501).

**Variable**	**aOR (95% CI)**	***p*-value**
**HbA1c level**	-	-
≥7%	1.9 (1.2-2.9)	0.003*
<7%	Reference	
**Comorbidity**	-	-
Yes	1.6 (1.1-2.4)	0.018*
No	Reference	
**Antidiabetics**	-	-
Insulin/sulfonylurea user	2.0 (1.0-4.0)	0.038*
Not insulin/sulfonylurea user	Reference	
**Smoking**	-	-
Yes	0.45 (0.28-0.77)	0.007*
No	Reference	
**Physical activity**	-	-
Active	1.8 (1.2-2.6)	0.002*
Less active	Reference	

**Note:** * = *p*<0.05, aOR = adjusted Odds Ratio.

## Data Availability

The data and supportive information are available within the article.

## LIST OF ABBREVIATIONS

PAH: Polycyclic Aromatic Hydrocarbon
DSME: Diabetes Self-Management Education
ADA: American Diabetes Association
DM: Diabetes Mellitus

## STANDARDS OF REPORTING

STROBE guidelines were followed.

## References

[1] (2021). Diabetes Atlas IDF. IDF Diabetes Atlas.

[2] (2019). Indonesian Ministry of Health National Health Research Report..

[3] Alkaff F.F., Illavi F., Salamah S. (2021). The impact of the indonesian chronic disease management program (PROLANIS) on metabolic control and renal function of type 2 diabetes mellitus patients in primary care setting.. J. Prim. Care Community Health.

[4] Khoe L.C., Wangge G., Soewondo P., Tahapary D.L., Widyahening I.S. (2020). The implementation of community-based diabetes and hypertension management care program in Indonesia.. PLoS One.

[5] Indonesian Ministry of Health (2021). Decree of the Minister of Health Number HK. In: 0107/MENKES/6485/2021 on the National Formulary..

[6] (2021). Indonesian endocrinology association guidelines for the management and prevention of type 2 diabetes mellitus in adults in Indonesia.. PB Perkeni.

[7] Davies M.J., D’Alessio D.A., Fradkin J. (2018). Management of hyperglycemia in type 2 diabetes, 2018. A consensus report by the american diabetes association (ADA) and the European association for the study of diabetes (EASD).. Diabetes Care.

[8] Annisa B.S., Puspitasari C.E., Aini S.R. (2021). Profil penggunaan obat antidiabetes pada pasien diabetes mellitus tipe 2 di instalasi rawat jalan RSUD Provinsi NTB tahun 2018.. Sasambo J Pharm.

[9] Binti Sappo N., Rahmawati D., Ramadhan A.M. Pharmaceutical research and development L, tropis F. characteristics and patterns of anti-diabetic drug use in type 2 diabetes mellitus patients at Abdul Wahab Sjarahranie Regional Hospital.. Proceeding of Mulawarman Pharmaceuticals Conferences.

[10] Eroglu N., Sabuncu N. (2021). The effect of education given to type 2 diabetic individuals on diabetes self-management and self-efficacy: Randomized controlled trial.. Prim. Care Diabetes.

[11] Norris S., Nichols P., Caspersen C. (2002). Increasing diabetes self-management education in community settings a systematic review.. Am. J. Prev. Med..

[12] Greenway F.L. (2016). Severe hypoglycemia in the look ahead trial.. J. Diabetes Complications.

[13] Iloh G.P., Amadi A. (2018). Epidemiology of hypoglycemia among ambulatory Type 2 diabetic patients in a primary care clinic of a tertiary hospital in Southeastern Nigeria.. J Health Res Rev.

[14] Shriraam V., Mahadevan S., Anitharani M. (2017). Reported hypoglycemia in Type 2 diabetes mellitus patients: Prevalence and practices-a hospital-based study.. Indian J. Endocrinol. Metab..

[15] Snell-Bergeon J.K., Wadwa R.P. (2012). Hypoglycemia, diabetes, and cardiovascular disease.. Diabetes Technol. Ther..

[16] Morales J., Schneider D. (2014). Hypoglycemia.. Am. J. Med..

[17] Besen DB, Surucu HA, Koşar C (2016). Self-reported frequency, severity of, and awareness of hypoglycemia in type 2 diabetes patients in Turkey PeerJ.

[18] van Meijel L.A., de Vegt F., Abbink E.J. (2020). High prevalence of impaired awareness of hypoglycemia and severe hypoglycemia among people with insulin-treated type 2 diabetes: The dutch diabetes pearl cohort.. BMJ Open Diabetes Res. Care.

[19] Hussein Z., Kamaruddin N.A., Chan S.P., Jain A., Uppal S., Bebakar W.M.W. (2017). Hypoglycemia awareness among insulin-treated patients with diabetes in Malaysia: A cohort subanalysis of the HAT study.. Diabetes Res. Clin. Pract..

[20] Bain A., Kavanagh S., McCarthy S., Babar Z.U.D. (2019). Assessment of insulin-related knowledge among healthcare professionals in a large teaching hospital in the united kingdom.. Pharmacy.

[21] Rezende P.C., Everett B.M., Brooks M.M. (2018). Hypoglycemia and elevated troponin in patients with diabetes and coronary artery disease.. J. Am. Coll. Cardiol..

[22] Heller S.R., Geybels M.S., Iqbal A., Liu L., Wagner L., Chow E. (2021). A higher non-severe hypoglycaemia rate is associated with an increased risk of subsequent severe hypoglycaemia and major adverse cardiovascular events in individuals with type 2 diabetes in the LEADER study.. Diabetologia.

[23] Echouffo-Tcheugui J.B., Daya N., Lee A.K. (2021). Severe hypoglycemia, cardiac structure and function, and risk of cardiovascular events among older adults with diabetes.. Diabetes Care.

[24] Quilliam B.J., Simeone J.C., Ozbay A.B., Kogut S.J. (2011). The incidence and costs of hypoglycemia in type 2 diabetes.. Am. J. Manag. Care.

[25] Aljunid S.M., Aung Y.N., Ismail A. (2019). Economic burden of hypoglycemia for type II diabetes mellitus patients in Malaysia.. PLoS One.

[26] Strizek A., Chang C.J., Furnback W., Wang B., Lebrec J., Lew T. (2019). The cost of hypoglycemia associated with type 2 diabetes mellitus in Taiwan.. Value Health Reg. Issues.

[27] Hidayat B., Ramadani R.V., Rudijanto A., Soewondo P., Suastika K., Siu Ng J.Y. (2022). Direct medical cost of type 2 diabetes mellitus and its associated complications in Indonesia.. Value Health Reg. Issues.

[28] Hall T.A. (1987). Designing culturally relevant educational materials for Mexican American clients.. Diabetes Educ..

[29] Park J.S., Ahn C.W. (2007). Educational program for diabetic patients in Korea—Multidisplinary intensive management.. Diabetes Res. Clin. Pract..

[30] LaManna J., Litchman M.L., Dickinson J.K. (2019). Diabetes education impact on hypoglycemia outcomes: A systematic review of evidence and gaps in the literature.. Diabetes Educ..

[31] Rudijanto A., Saraswati M.R., Yunir E., Kumala P., Puteri H.H.S., Mandang V.V.V. (2018). Indonesia cohort of IO HAT study to evaluate diabetes management, control, and complications in retrospective and prospective periods among insulin-treated patients with type 1 and type 2 diabetes.. Acta Med. Indones..

[32] Seaquist E.R., Anderson J., Childs B. (2013). Hypoglycemia and diabetes: A report of a workgroup of the American Diabetes Association and the Endocrine Society.. Diabetes Care.

[33] Rickels M.R. (2019). Hypoglycemia‐associated autonomic failure, counterregulatory responses, and therapeutic options in type 1 diabetes.. Ann. N. Y. Acad. Sci..

[34] Mohseni M., Ahmadi S., Azami-Aghdash S. (2021). Challenges of routine diabetes care during COVID-19 era: A systematic search and narrative review.. Prim. Care Diabetes.

[35] Parkin C.G., Davidson J.A. (2009). Value of self-monitoring blood glucose pattern analysis in improving diabetes outcomes.. J. Diabetes Sci. Technol..

[36] Klonoff D.C. (2013). The current status of Health for diabetes: Will it be the next big thing?. J. Diabetes Sci. Technol..

[37] Kacheva S., Karges B., Göller K., Marx N., Mischke K., Karges W. (2017). QT prolongation caused by insulin-induced hypoglycaemia – An interventional study in 119 individuals.. Diabetes Res. Clin. Pract..

[38] Pearson S.M., Whittam B., Kulavarasalingam K., Mitchell-Gears A., James C., Ajjan R.A. (2021). Reduction in cardiovascular mortality following severe hypoglycemia in individuals with type 2 diabetes: The role of a pragmatic and structured intervention.. Cardiovasc. Diabetol..

[39] Lamounier R.N., Geloneze B., Leite S.O. (2018). Hypoglycemia incidence and awareness among insulin-treated patients with diabetes: The HAT study in Brazil.. Diabetol. Metab. Syndr..

[40] Borzì V., Frasson S., Gussoni G. (2016). Risk factors for hypoglycemia in patients with type 2 diabetes, hospitalized in internal medicine wards: Findings from the FADOI-DIAMOND study.. Diabetes Res. Clin. Pract..

[41] Avari P., Moscardo V., Jugnee N., Oliver N., Reddy M. (2020). Glycemic variability and hypoglycemic excursions with continuous glucose monitoring compared to intermittently scanned continuous glucose monitoring in adults with highest risk type 1 diabetes.. J. Diabetes Sci. Technol..

[42] Malkani S, Kotwal A (2017). Frequency and predictors of self-reported hypoglycemia in insulin-treated diabetes.. J Diabetes Res.

[43] Lee A.K., Lee C.J., Huang E.S., Sharrett A.R., Coresh J., Selvin E. (2017). Risk factors for severe hypoglycemia in black and white adults with diabetes: The atherosclerosis risk in communities (ARIC) study.. Diabetes Care.

[44] Akirov A., Amitai O., Masri-Iraqi H. (2018). Predictors of hypoglycemia in hospitalized patients with diabetes mellitus.. Intern. Emerg. Med..

[45] Au N.H., Ratzki-Leewing A., Zou G., Ryan B.L., Webster-Bogaert S., Reichert S.M. (2021). Real-world incidence and risk factors for daytime and nocturnal non-severe hypoglycemia in adults with type 2 diabetes mellitus on insulin and/or secretagogues (InHypo-DM Study, Canada).. Can. J. Diabetes.

[46] Nuzzo A., Brignoli A., Chantal Ponziani M., Zavattaro M., Prodam F., Castello L.M. (2021). Aging and comorbidities influence the risk of hospitalization and mortality in diabetic patients experiencing severe hypoglycemia.. Nutr. Metab. Cardiovasc. Dis..

[47] Lv X., Fang K., Hao W., Han Y., Yang N., Yu Q. (2020). Identification of reactive hypoglycemia with different basic bmi and its causes by prolonged oral glucose tolerance test.. Diabetes Metab. Syndr. Obes..

[48] Bie-Olsen L.G., Pedersen-Bjergaard U., Kjær T.W., Lonsdale M.N., Law I., Thorsteinsson B. (2010). Differences in cortical and pituitary activity in response to hypoglycaemia and cognitive testing in healthy men with different basal activity of the renin-angiotensin system.. J. Renin Angiotensin Aldosterone Syst..

[49] Ensling M., Steinmann W., Whaley-Connell A. (2011). Hypoglycemia: A possible link between insulin resistance, metabolic dyslipidemia, and heart and kidney disease (the Cardiorenal Syndrome).. Cardiorenal Med..

[50] Pratiwi C., Mokoagow M.I., Made Kshanti I.A., Soewondo P. (2020). The risk factors of inpatient hypoglycemia: A systematic review.. Heliyon.

[51] Ray C.Y., Wu V.C.C., Wang C.L. (2021). Hypoglycemia associated with drug–drug interactions in patients with type 2 diabetes mellitus using dipeptidylpeptidase-4 inhibitors.. Front. Pharmacol..

[52] Romley J.A., Gong C., Jena A.B., Goldman D.P., Williams B., Peters A. (2015). Association between use of warfarin with common sulfonylureas and serious hypoglycemic events: Retrospective cohort analysis.. BMJ.

[53] Thamer M., Ray N.F., Taylor T. (1999). Association between antihypertensive drug use and hypoglycemia: A case-control study of diabetic users of insulin or sulfonylureas.. Clin. Ther..

[54] Parekh T.M., Raji M., Lin Y.L., Tan A., Kuo Y.F., Goodwin J.S. (2014). Hypoglycemia after antimicrobial drug prescription for older patients using sulfonylureas.. JAMA Intern. Med..

[55] Sonoda N., Morimoto A., Ugi S. (2015). Predictors for mild and severe hypoglycemia in insulin-treated Japanese diabetic patients.. PLoS One.

[56] Nassar D.T., Habib O.S., Mansour A.A. (2016). Predictors of hypoglycemia in insulin-treated patients with type 2 diabetes mellitus in Basrah.. World J. Diabetes.

[57] Ajjan R.A., Jackson N., Thomson S.A. (2019). Reduction in HbA1c using professional flash glucose monitoring in insulin-treated type 2 diabetes patients managed in primary and secondary care settings: A pilot, multicentre, randomised controlled trial.. Diab. Vasc. Dis. Res..

[58] Cuddihy R.M., Philis-Tsimikas A., Nazeri A. (2011). Type 2 diabetes care and insulin intensification: Is a more multidisciplinary approach needed? Results from the MODIFY survey.. Diabetes Educ..

[59] Gómez-Huelgas R., Sabán-Ruiz J., García-Román F.J. (2017). Safety and efficacy of a basal-plus regimen with insulin glargine and insulin glulisine for elderly patients with high cardiovascular risk and type 2 diabetes mellitus.. Rev Clin Esp.

[60] Rados D.V., Falcetta M.R.R., Pinto L.C., Leitão C.B., Gross J.L. (2021). All-cause mortality and cardiovascular safety of basal insulin treatment in patients with type 2 diabetes mellitus: A systematic review with meta-analysis and trial sequential analysis.. Diabetes Res. Clin. Pract..

[61] Mannucci E., Targher G., Nreu B. (2022). Effects of insulin on cardiovascular events and all-cause mortality in patients with type 2 diabetes: A meta-analysis of randomized controlled trials.. Nutr. Metab. Cardiovasc. Dis..

[62] Li Z., Zhang Y., Quan X. (2016). Efficacy and acceptability of glycemic control of glucagon-like peptide-1 receptor agonists among type 2 diabetes: A systematic review and network meta-analysis.. PLoS One.

[63] Xu X., Xu W., Zhuo Q., Yan Y. (2022). The efficacy and safety of dapagliflozin combined with oral hypoglycemic agents in patients with type 2 diabetes: A systematic review and meta-analysis.. Ann. Palliat. Med..

[64] Zhao J.Z., Weinhandl E.D., Carlson A.M., St Peter W.L. (2022). Hypoglycemia risk with SGLT2 inhibitors or glucagon-like peptide 1 receptor agonists versus sulfonylureas among medicare insured adults with CKD in the United States.. Kidney Med..

[65] Sari M.I., Sari N., Darlan D.M., Prasetya R.J. (2018). Cigarette smoking and hyperglycaemia in diabetic patients.. Open Access Maced. J. Med. Sci..

[66] Duarte C.K., Almeida J.C., Merker A.J., Brauer FdeO, Rodrigues TdaC (2012). Physical activity level and exercise in patients with diabetes mellitus.. Rev. Assoc. Med. Bras..

[67] Castonguay A., Miquelon P., Boudreau F. (2018). Self-regulation resources and physical activity participation among adults with type 2 diabetes.. Health Psychol. Open.

[68] Zhu P., Pan X.F., Sheng L., Chen H., Pan A. (2017). Cigarette smoking, diabetes, and diabetes complications: Call for urgent action.. Curr. Diab. Rep..

[69] Eliasson B. (2003). Cigarette smoking and diabetes.. Prog. Cardiovasc. Dis..

[70] Artese A., Stamford B.A., Moffatt R.J. (2019). Cigarette smoking: An accessory to the development of insulin resistance.. Am. J. Lifestyle Med..

[71] Campagna D., Alamo A., Di Pino A., Russo C., Calogero A.E., Purrello F. (2019). Smoking and diabetes: Dangerous liaisons and confusing relationships.. Diabetol Metab.

[72] Maddatu J., Anderson-Baucum E., Evans-Molina C. (2017). Smoking and the risk of type 2 diabetes.. Transl. Res..

[73] Maideen NMP Tobacco smoking and its drug interactions with comedications involving CYP and UGT enzymes and nicotine.

[74] Porter J.W., Pettit-Mee R.J., Ready S.T. (2020). Post meal exercise may lead to transient hypoglycemia irrespective of glycemic status in humans.. Front. Endocrinol..

[75] Younk L.M., Mikeladze M., Tate D., Davis S.N. (2011). Exercise-related hypoglycemia in diabetes mellitus.. Expert Rev. Endocrinol. Metab..

